# Aneuploidy Can Be an Evolutionary Diversion on the Path to Adaptation

**DOI:** 10.1093/molbev/msae052

**Published:** 2024-03-01

**Authors:** Ilia Kohanovski, Martin Pontz, Pétra Vande Zande, Anna Selmecki, Orna Dahan, Yitzhak Pilpel, Avihu H Yona, Yoav Ram

**Affiliations:** School of Zoology, Faculty of Life Sciences, Tel Aviv University, Tel Aviv, Israel; School of Computer Science, Reichman University, Herzliya, Israel; School of Zoology, Faculty of Life Sciences, Tel Aviv University, Tel Aviv, Israel; Department of Microbiology and Immunology, University of Minnesota Medical School, Minneapolis, MN, USA; Department of Microbiology and Immunology, University of Minnesota Medical School, Minneapolis, MN, USA; Department of Molecular Genetics, Weizmann Institute of Science, Rehovot, Israel; Department of Molecular Genetics, Weizmann Institute of Science, Rehovot, Israel; Institute of Biochemistry, Food Science and Nutrition, Robert H. Smith Faculty of Agriculture, Food and Environment, The Hebrew University of Jerusalem, Rehovot, Israel; School of Zoology, Faculty of Life Sciences, Tel Aviv University, Tel Aviv, Israel

**Keywords:** whole-chromosome duplication, evolutionary model, adaptive evolution

## Abstract

Aneuploidy is common in eukaryotes, often leading to decreased fitness. However, evidence from fungi and human tumur cells suggests that specific aneuploidies can be beneficial under stressful conditions and facilitate adaptation. In a previous evolutionary experiment with yeast, populations evolving under heat stress became aneuploid, only to later revert to euploidy after beneficial mutations accumulated. It was therefore suggested that aneuploidy is a “stepping stone” on the path to adaptation. Here, we test this hypothesis. We use Bayesian inference to fit an evolutionary model with both aneuploidy and mutation to the experimental results. We then predict the genotype frequency dynamics during the experiment, demonstrating that most of the evolved euploid population likely did not descend from aneuploid cells, but rather from the euploid wild-type population. Our model shows how the beneficial mutation supply—the product of population size and beneficial mutation rate–determines the evolutionary dynamics: with low supply, much of the evolved population descends from aneuploid cells; but with high supply, beneficial mutations are generated fast enough to outcompete aneuploidy due to its inherent fitness cost. Our results suggest that despite its potential fitness benefits under stress, aneuploidy can be an evolutionary “diversion” rather than a “stepping stone”: it can delay, rather than facilitate, the adaptation of the population, and cells that become aneuploid may leave less descendants compared to cells that remain diploid.

## Introduction

Aneuploidy is an imbalance in the number of chromosomes in the cell: an incorrect karyotype. Evidence suggests that aneuploidy is very common in eukaryotes, e.g. animals (Santaguida and Amon 2015; Naylor and van Deursen 2016; Bakhoum and Landau 2017) and fungi (Pavelka et al. 2010; Zhu et al. 2016; Robbins et al. 2017; Todd et al. 2017). Aneuploidy has been implicated in cancer formation, progression, and drug resistance (Boveri 2008; Schvartzman et al. 2010; Santaguida and Amon 2015; Rutledge et al. 2016; Ippolito et al. 2021; Lukow et al. 2021). It is also common in protozoan pathogens of the Leishmania genus, a major global health concern (Mannaert et al. 2012), and contributes to the emergence of drug resistance (Selmecki et al. 2009) and virulence (Möller et al. 2018) in fungal pathogens, which are under-studied (Rodrigues and Albuquerque 2018), despite infecting a billion people per year, causing significant morbidity in >150 million and death in >1.5 million people per year (Selmecki et al. 2009; Rodrigues and Albuquerque 2018).

Experiments with human and mouse embryos found that most germ-line aneuploidies are lethal. Aneuploidies are also associated with developmental defects and lethality in other multicellular organisms (Sheltzer and Amon 2011). For example, aneuploid mouse embryonic cells grow slower than euploid cells (Williams et al. 2008). Similarly, in unicellular eukaryotes growing in benign conditions, aneuploidy usually leads to slower growth and decreased overall fitness, in part due to proteotoxic stress due to increased expression, gene dosage imbalance, and hypo-osmotic-like stress (Niwa et al. 2006; Torres et al. 2007; Pavelka et al. 2010; Sheltzer and Amon 2011; Santaguida et al. 2015; Kasuga et al. 2016; Zhu et al. 2018; Tsai et al. 2019; Yang et al. 2021; Robinson et al. 2023).

However, aneuploidy can be beneficial under stressful conditions due to the wide range of phenotypes it can produce, some of which are advantageous (Pavelka et al. 2010; Yang et al. 2021). Indeed, in a survey of 1,011 yeast strains, aneuploidy has been detected in about 19% (Peter et al. 2018). Thus, aneuploidy can lead to rapid adaptation in unicellular eukaryotes (Rancati et al. 2008; Torres et al. 2010; Hong and Gresham 2014; Gerstein et al. 2015), as well as to rapid growth of somatic tumor cells (Schvartzman et al. 2010; Sheltzer et al. 2017). For example, aneuploidy in *Saccharomyces cerevisiae* facilitates adaptation to a variety of stressful conditions like heat and pH (Yona et al. 2012), copper (Covo et al. 2014; Gerstein et al. 2015), salt (Dhar et al. 2011; Robinson et al. 2023), and nutrient limitation (Dunham et al. 2002; Gresham et al. 2008; Avecilla et al. 2022), with similar results in *Candida albicans* (Yang et al. 2021). Importantly, aneuploidy can also lead to drug resistance in pathogenic fungi such as *C. albicans* (Selmecki et al. 2008, 2010; Gerstein and Berman 2020) and *Cryptococcus neoformans* (Sionov et al. 2010), which cause candidiasis and meningoencephalitis, respectively. Although we focus here on aneuploidy, a similar phenomena of adaptation via gene duplication or amplification has been observed in yeast (Lauer et al. 2018), bacteria (Sonti and Roth 1989), and DNA viruses (Elde et al. 2012).

Yona et al. (2012) demonstrated experimentally the importance of aneuploidy in adaptive evolution. They evolved populations of *S. cerevisiae* under strong heat stress. The populations adapted to the heat stress within 450 generations, and this adaptation was determined to be due a duplication of chromosome III. Later on, after more than 1,500 generations, the populations reverted back to an euploid state, while remaining adapted to the heat stress. Aneuploidy was therefore suggested to be a transient adaptive solution, because it can rapidly appear and take over the population under stressful conditions, and can then be rapidly lost when the cost of aneuploidy outweighs its benefit—after the stress is removed, or after refined beneficial mutations appear and fix (Yona et al. 2012). Furthermore, it has been suggested that aneuploidy is an evolutionary “stepping stone” that facilitates future adaptation by genetic mutations, which require more time to evolve (Yona et al. 2012, 2015).

Here, we test the hypothesis that aneuploidy is an evolutionary “stepping stone” that facilitates adaptive evolution by genetic mutations (Yona et al. 2012). We develop an evolutionary genetic model and fit it to the experimental results of Yona et al. (2012) to predict the genotype frequency dynamics in the experimental populations, thereby estimating the frequency of evolved euploid cells that descended from aneuploid cells. Our results show that although aneuploidy reached high frequencies in the experimental populations, the majority of cells in the evolved euploid population likely did not descend from aneuploid cells, but rather directly from wild-type euploid cells. These suggest that at the lineage level, aneuploidy may be an “evolutionary diversion”, rather than a “stepping stone”, on the path to adaptation.

## Results

In the heat-stress experiment of Yona et al. (2012), four populations of *S. cerevisiae* evolved under 39 °C. Aneuploidy reached high frequency (>95%) in all four experimental repetitions in the first 450 generations. Two of the repetitions, marked H2 and H4, carried no large-scale duplications other than a chromosome III trisomy. These two repetitions continued to evolve under the same conditions, wherein aneuploidy was eliminated by generation 1,700 and 2,350 in H4 and H2, respectively.

###  

####  

##### Evolutionary genetic model

To explore the dynamics during the evolutionary experiments, we developed an evolutionary genetic model, fitted the model to empirical data, and used it to predict the genotype frequency dynamics, or specifically, the fraction of the evolved euploid population descended from aneuploid cells.

**Fig. 1.**
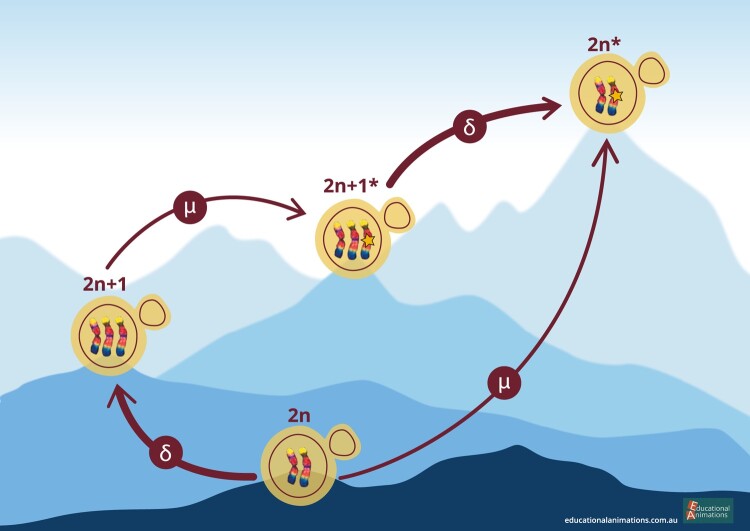
Model illustration. There are four genotypes in our model: euploid wild-type, 2n; euploid mutant, $2n^{*}$; aneuploid wild-type, 2n+1; and aneuploid mutant, $2n+1^{*}$. Overall there are two possible trajectories from 2n to $2n^{*}$. Arrows denote transitions between genotypes, with transition rates *μ* for the beneficial mutation rate and *δ* for the aneuploidy rate. Elevation differences illustrate the expected, rather than the assumed, fitness differences between the genotypes.

The model includes the effects of natural selection, genetic drift, aneuploidy, and mutation (i.e. other genetic variants) and follows a population of cells characterized by their genotype: euploid wild-type, 2n, is the ancestral diploid genotype; euploid mutant, $2n^{*}$, has a diploid karyotype and a single beneficial mutation; aneuploid wild-type, 2n+1, has an extra chromosome due to a chromosome duplication event; and aneuploid mutant, $2n+1^{*}$, has an extra chromosome (like 2n+1) and a beneficial mutation (like $2n^{*}$). Note that “mutation” here refers to point mutations and other genetic variants unrelated to aneuploidy. Fitness values of the different genotypes are denoted by $w_{2n}$, $w_{2n^{*}}$, $w_{2n+1}$, and $w_{2n+1^{*}}$, and the rate of mutation and aneuploidy are denoted by *μ* and *δ*, respectively. See Fig. 1 for an illustration of the model.

We fitted this model to the experimental results (Yona et al. 2012)—time for fixation (frequency >95%) and for loss (frequency <5%) of aneuploidy—using approximate Bayesian computation with sequential Monte-Carlo (ABC-SMC; Sisson et al. 2007), thereby inferring the model parameters: rates of aneuploidy (i.e. mis-segregation, non-disjunction) and mutation and the fitness of all genotypes. We then sampled posterior predictions for the genotype frequency dynamics using the estimated parameter values and compared different versions of the model to test additional hypotheses about the evolutionary process.

##### Estimated rates and fitness effects of aneuploidy and mutation

**Fig. 2.**
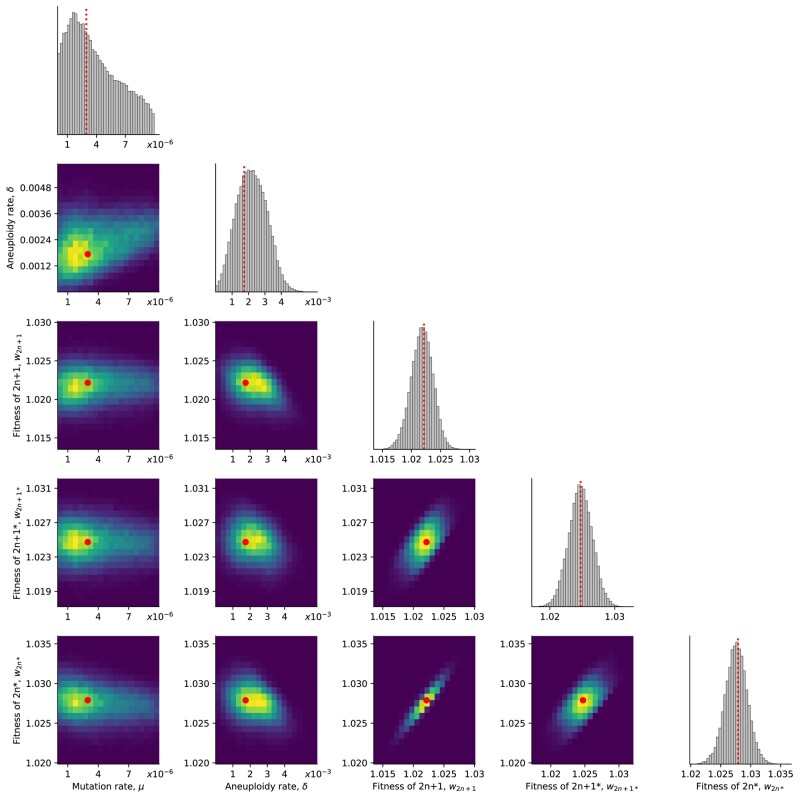
Posterior distribution of model parameters. On the diagonal, the marginal posterior distribution of each model parameter. Below the diagonal, the joint posterior distribution of pairs of model parameters (dark and bright for low and high density, respectively). Markers and lines for the joint MAP estimate (which may differ from the marginal MAP, as the marginal distribution integrates over all other parameters).

We inferred the posterior distribution of model parameters (Fig. 2). We report parameter estimates using the maximum a posteriori (MAP) and providing the 50% highest density interval (HDI) in square brackets. See Supplementary material online for sensitivity analysis.

The estimated beneficial mutation rate is $\mu =2.965\cdot 10^{-6}\;[2.718\cdot 10^{-7}-3.589\cdot 10^{-6}]$ per genome per generation (that is, roughly three out of $10^{6}$ cell divisions produce a mutant cell with a fitness advantage). From the literature, the mutation rate per base pair is roughly $2\text{–}3\cdot 10^{-10}$ (Lynch et al. 2008; Zhu et al. 2014), but it may be higher under heat stress, as several stresses (Heidenreich 2007), including heat (Huang et al. 2018), may cause hypermutation in yeast. If we assume a 10-fold increase over the mutation rate reported in the literature, then the estimated beneficial mutation rate can be explained by a genomic target size of 1,000 base pairs (that is, 1,000 base pairs across the genome in which a mutation would provide a fitness advantage): $3\cdot 10^{-10}\times 10\times 1,000=3\cdot 10^{-6}$. Supporting this, Jarolim et al. (2013) found 279 genes that contributed to survival after a sudden shift from 30 °C to 50 °C, and Flynn et al. (2020) used a deep mutational scan of a single protein, Hsp90, to find 465 amino-acid variants (out of 14,160) that significantly increased growth rate in 37 °C. Furthermore, Yona et al. (2012) found at least 10 genes on chromosome III that increased heat tolerance when over-expressed. Assuming that other chromosomes also have a similar number of heat-tolerance genes (and even more, as chromosome III is one of the smallest chromosomes; Gilchrist and Stelkens 2019), we estimate a total of 160 heat-tolerance genes in the genome. Indeed, mutations were found in 97 genes in an evolutionary experiment with yeast under heat stress (Huang et al. 2018). Thus, to get a genomic target size of 1,000, it is enough that the average gene target size (number of base pairs in a gene in which a mutation is beneficial) is 6.25 base pairs. For example, Kohn and Anderson (2014) found a target size of 11 in a proton exporter gene (*PMA1*) that contributes to high-salt adaptation.

The estimated rate of aneuploidy (i.e. mis-segregation, non-disjunction), $\delta =1.72\cdot 10^{-3}\;[1.47\cdot 10^{-3}$–$2.786\cdot 10^{-3}]$, is higher than in previous studies: for chromosome III in diploid *S. cerevisiae*, Zhu et al. (2014) estimated $6.7\cdot 10^{-6}$ chromosome gain events per generation, and Kumaran et al. (2013) estimate $3.0\cdot 10^{-5}$–$4.3\cdot 10^{-5}$ chromosome loss events per generation (95% confidence interval). However, this difference may be partly explained by an increased rate of aneuploidy during heat stress: heat shock can increase the rate of chromosome fragment loss by 2–3 orders of magnitude (Chen et al. 2012).

The estimated fitness values are $w_{2n+1}=1.022$  [1.021– 1.023], $w_{2n+1^{*}}=1.025\;[1.024$–1.026], and $w_{2n^{*}}=1.028$  [1.026–1.029], all relative to the fitness of 2n, which is set to $w_{2n}=1$. If we allow for transitions (mutation, chromosome loss and gain) to less-fit genotypes (e.g. $2n^{*}$ to $2n+1^{*}$), then we infer similar but slightly different values, see Supplementary material online.

##### Model comparison and goodness-of-fit

**Fig. 3.**
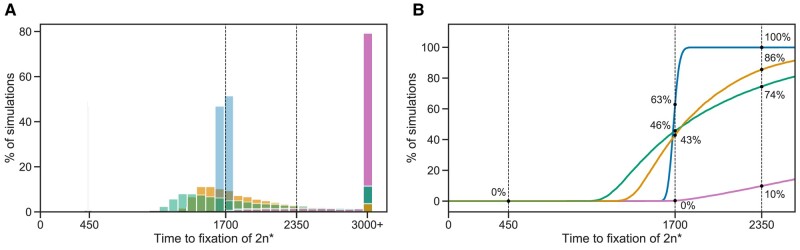
Model fit with and without aneuploidy. The distribution of time to fixation of $2n^{*}$ (i.e. adaptation time) in 10,000 simulations using MAP parameters of the model with beneficial aneuploidy (blue; $\delta >0,\;w_{2n}<w_{2n+1}<w_{2n+1^{*}}<w_{2n^{*}}$) compared to alternative models: a model with the same parameter values but without aneuploidy (gray, δ=0, concentrated at t=450); a model fitted to the data assuming no aneuploidy (green, δ=0); a model fitted to the data assuming neutral aneuploidy (yellow, $\delta >0,\;w_{2n+1}=w_{2n},\;w_{2n+1^{*}}=w_{2n^{*}}$); and a model with beneficial aneuploidy and an extended prior distribution (pink). In the experiment by Yona et al. (2012), one population lost aneuploidy by generation 1,700 and another by generation 2,350 (dashed lines) but not before generation 450. Thus, the blue distribution has a better fit compared to the other distributions (the gray distribution has a particularly poor fit). The MAP likelihood (equation 4) is 0.84, 0.78, 0.67, and 0.14 for the models represented by blue, yellow, green, and pink distributions, respectively. A) Histogram of the time to fixation of $2n^{*}$. The last bin contains all values equal or greater than 3,000. B) Cumulative distribution of the time to fixation.

To assess the fit of our model to the data, we use posterior predictive checks, in which we simulate the frequency dynamics using MAP parameter estimates and compare them to the data. Our model fits the data well: $2n^{*}$ fixed in 63% of simulations by generation 1,700 and in 100% of simulations by generation 2,350 (Fig. 3).

However, a model without aneuploidy (where the aneuploidy rate is fixed at zero, δ=0) fails to explain the experimental observations (Fig. 3). The estimated mutation rate without aneuploidy is $\mu =7.98\cdot 10^{-9}\;[7.906\cdot 10^{-9}$–$8.138\cdot 10^{-9}]$, much lower compared to a model with aneuploidy. The fitness of the mutant is also much lower at $w_{2n^{*}}=1.013$  [1.012–1.013]. This is because, without aneuploidy, a high mutation rate or fitness effect will lead to faster appearance and fixation of $2n^{*}$ than in the experimental observations.

We also checked a model in which aneuploidy occurs but is adaptively neutral compared to the wild-type, that is, $w_{2n+1}=w_{2n}$ and $w_{2n+1^{*}}=w_{2n^{*}}$ but δ>0. This model fits the data better than the model with no aneuploidy (in which δ=0), but worse than a model with positive selection for aneuploidy, in which $w_{2n}<w_{2n+1}<w_{2n+1^{*}}$  $<w_{2n^{*}}$ (Fig. 3).

##### Model predictions of genotype frequency dynamics

**Fig. 4.**
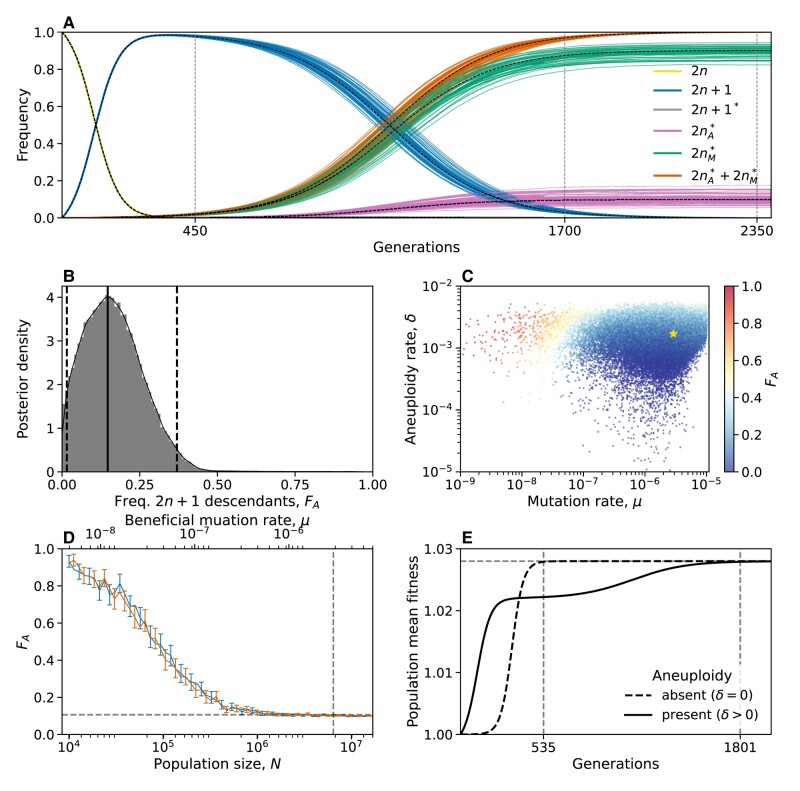
Predicted frequency of aneuploid-descended cells. A) Posterior predicted genotype frequencies over time, including the source of $2n^{*}$: $2n_{A}^{*}$ arose from 2n+1, whereas $2n_{M}^{*}$ arose directly from 2n. Colored curves are 50 simulations using the MAP estimate parameters. Black dashed curves are the expected genotype frequencies without genetic drift (from a deterministic model). See supplementary fig. S9, Supplementary Material online, for log–log scale, in which the sequence of events is easier to observe. B) Posterior distribution of $F_{A}$, the expected frequency of $2n^{*}$ cells descended from 2n+1 cells, computed as the average frequency at the end of 100 simulations for 100,000 samples from the parameter posterior distribution. Solid and dashed lines show the mode and 95% CI. C) $F_{A}$ values (color coded) from panel B, with their corresponding mutation rate *μ* on *x*-axis and aneuploidy rate *δ* on the *y*-axis. Yellow star shows the MAP estimate. See also supplementary fig. S10, Supplementary Material online. D) $F_{A}$ as a function of the population size (*N*, bottom *x*-axis) and the beneficial mutation rate (*μ*, top *x*-axis) in posterior predictions with MAP parameters. Markers show $F_{A}$ in 250 simulations per population size or mutation rate value. Error bars show mean $F_{A}$ with 95% CI (bootstrap, n=10,000). Blue and red bars for varying population size and mutation rate, respectively. Vertical dashed line for population size in the experiment, $6.425\cdot 10^{6}$, and the MAP mutation rate, $2.965\cdot 10^{-6}$. Horizontal line for $F_{A}^{\mathrm{MAP}}=0.106$. E) Population mean fitness in a model without drift using MAP estimate parameters. Solid lines for mean fitness with aneuploidy (δ>0), where the population reaches adaptation (mean fitness at 99.99% of maximum value) at generation 1,802. Dashed lines for mean fitness without aneuploidy (δ=0), where the population adapts much earlier, at generation 535.

We simulated 50 replicate genotype frequency dynamics using the MAP estimate parameters. Figure 4A shows the simulated frequencies of the four genotypes (2n, 2n+1, $2n+1^{*}$, and $2n^{*}$), as well as the frequencies of $2n^{*}$ cells that arose from either 2n+1 cells via a sequences of mutation and chromosome loss events ($2n_{A}^{*}$) or directly from 2n cells via a mutation event ($2n_{M}^{*}$). We find that $2n+1^{*}$ never reaches substantial frequency as it is quickly replaced by $2n^{*}$ in a process similar to *stochastic tunneling* (Komarova et al. 2003; Iwasa et al. 2004).

To test the hypothesis that aneuploidy facilitates adaptation, we estimated $F_{A}$, the expected frequency of $2n^{*}$ that arose from 2n+1, computed as the average frequency of such $2n_{A}^{*}$ cells at the end of simulations using the MAP estimate parameters. Surprisingly, we observe that the majority of $2n^{*}$ cells are $2n_{M}^{*}$, a product of a direct mutation in 2n cells, rather than descending from 2n+1 cells ($F_{A}^{\mathrm{MAP}}=0.106$, average end point of 50 purple lines in Fig. 4A). This is despite the fact that the 2n+1 genotype reaches high frequencies in the population (at least 0.98; Fig. 4A).

This result is not unique to the MAP parameter estimate. We simulated genotype frequency dynamics using parameter samples from the posterior distribution, and computed the posterior distribution of $F_{A}$ (Fig. 4B). The posterior mode $F_{A}$ was just 0.147 [0.0154–0.370 95% CI] and only in 489 of 100,000 posterior samples (0.489%), $F_{A}$ was larger than 0.5 (see Supporting Material for results when transitions to less-fit genotypes are allowed, such as $2n^{*}$ to $2n+1^{*}$). Thus, if we sample a random cell from the evolved $2n^{*}$ population, it is more likely to have descended directly from an euploid cell than from an aneuploid cell. The probability of $2n^{*}$ descending from 2n+1 ($F_{A}$) increases with the aneuploidy rate, *δ*, and decreases with both the population size *N* and the mutation rate, *μ* (Fig. 4C and D). In some cases it can also be affected by the fitness parameters (supplementary fig. S10, Supplementary Material online).

##### Genetic instability in aneuploid cells

It has been suggested that aneuploidy increases genomic instability: Sheltzer et al. (2011) have demonstrated a fold increase of between 2.2 and 7.1 in the mutation rate of disomic yeast (rather than trisomic yeast, the focus of our analysis). Therefore, we inferred model parameters under the assumption that the mutation rate increases in aneuploid cells by a factor τ=1, 33/32 (due to an additional chromosome), 2, 5, 10, or 100 (due to genetic instability). We found that the posterior distribution was similar for τ=1, 33/32, 2, and 5 (supplementary fig. S4, Supplementary Material online). Furthermore, we computed the WAIC (widely applicable information criterion), a criterion for model selection (see Methods). The WAIC values were similar for all *τ* values (supplementary table S1, Supplementary Material online).

Assuming a strong increase of the mutation rate in aneuploid cells, i.e. τ=100, the inferred mutation rate was $\mu =4.094\cdot 10^{-7}\;[6.252\cdot 10^{-8}$– $6.046\cdot 10^{-7}]$, and the inferred aneuploidy rate was $\delta =0.744\cdot 10^{-3}\;[0.506\cdot 10^{-3}$–$1.827\cdot 10^{-3}]$. Compared to inference made assuming no effect of aneuploidy on the mutation rate, these rates were about 7–8-fold and 2–3-fold lower for *μ* and *δ*, respectively. Assuming τ=10, the inferred mutation rate was only slightly lower compared to τ=1 ($\mu =1.67\cdot 10^{-6}$  $[2.836\cdot 10^{-8}$– $2.245\cdot 10^{-6}]$).

Therefore, we do not find evidence of an increase in mutation rate in aneuploid cells. This may be because, unless the increase is strong (τ≥10), it does not seem to affect our inference or because chromosome III is one of the smallest chromosomes (Gilchrist and Stelkens 2019). We also checked the differences in genotype frequency dynamics for different *τ* values. We observe that τ=100 could be distinguished if accurate data were available for the waiting time for the frequency of 2n to decrease below 95% (supplementary fig. S5A, Supplementary Material online) or for the waiting time for the frequency of 2n+1 to either reach or go below 95% (supplementary fig. S5B, Supplementary Material online).

Similarly, we did not find evidence for an increase in the rate of chromosome loss in aneuploid cells (Sheltzer et al. 2011), probably due to lack of statistical power. Nevertheless, increasing the rate of chromosome loss (transitions from $2n+1^{*}$ to $2n^{*}$) without increasing the rate of chromosome gain (transitions from 2n to 2n+1) increases $F_{A}$ (supplementary fig. S11B, Supplementary Material online), but not to the same extent as increasing the rate of chromosome gain (supplementary fig. S10, Supplementary Material online). In contrast, increasing the mutation rate in aneuploid cells can have a marked effect on the dynamics: when using the MAP parameter estimates, $F_{A}$ increases from 0.1 to 0.52 when the mutation rate in aneuploid cells increases 10-fold (supplementary fig. S11C, Supplementary Material online).

## Discussion

In a study on the role of chromosome duplication in adaptive evolution, Yona et al. (2012) found that a chromosome III trisomy was acquired by *S. cerevisiae* populations evolving under heat stress, only to be later replaced by euploid mutant cells that carry “refined” solutions to the stress. Additionally, such a replacement also occurred when they initiated evolutionary experiments with a population in which all cells carry a chromosome III trisomy. They hypothesized that aneuploidy is a “useful yet short-lived intermediate that facilitates further adaptation”, suggesting that the euploid mutant cells evolved by heat-resistance mutations in aneuploid cells followed by reversion of trisomy due to a chromosome loss event.

We developed an evolutionary genetic model of adaptive evolution by aneuploidy and mutation (Fig. 1), fitted it to the experimental results of Yona et al. (2012), and used it to predict the genotype frequency dynamics. The model predicted that only about 10–15% of the evolved euploid population descended from aneuploid cells by acquiring a mutation and losing the extra chromosome—that is, the majority of the euploid population are not descended from aneuploid cells, but rather are direct descendants of the ancestral wild-type population (Fig. 4).

This happens despite aneuploidy reaching a high frequency in the population (>95%). Conventional wisdom might suggest that once the aneuploid genotype 2n+1 reaches high frequency, it will have a better chance at producing “refined” solutions via mutations, and its descendants will come to dominate the population: the frequency of $2n_{A}^{*}$ (which arises from $2n+1^{*}$) will be higher than the frequency of $2n_{M}^{*}$ (which arises directly from 2n).

So how does $2n_{M}^{*}$ prevail? Initially, the supply rates of 2n+1 and $2n_{M}^{*}$ are Nδ≈11,000 and Nμ≈19, respectively (assuming MAP parameter estimates). Therefore, both genotypes are expected to appear immediately at the beginning of the experiment (supplementary fig. S9, Supplementary Material online). However, 2n+1 appears at a much higher frequency as δ≫μ by 2–3 orders of magnitude. After they first appear, $2n_{M}^{*}$ has higher fitness. But as long as the frequency of 2n is high, the supply rate of 2n+1 is higher than that of $2n_{M}^{*}$, again due to δ≫μ. However, supply rates of both genotypes decrease with the frequency of 2n. Therefore, when the latter decreases, mainly due to the increase in the frequency of 2n+1, both supply rates diminish. At this stage, the higher fitness of $2n_{M}^{*}$ comes into play and it starts to take over the population, which is mainly composed of 2n+1. For the aneuploid lineage to compete with the mutant lineage, it must produce $2n_{A}^{*}$ via a mutation followed by chromosome loss. Although this is a stochastic process (due to drift), our results show that the time until $2n_{A}^{*}$ reaches a frequency of 0.1% is roughly 450 generations, without much variation (intersection of purple lines and vertical dashed line in supplementary fig. S9, Supplementary Material online). However, by that time, $2n_{M}^{*}$ is already at a roughly 10-fold higher frequency (1.86%), and since both mutants have the same fitness, their relative frequency remains roughly the same until the end of the experiment.

###  

####  

##### Predictions for small populations and low mutation rates

We examined the effect of the population size, *N*, and the beneficial mutation rate, *μ*, on the frequency of 2n+1 descendants in the evolved population, $F_{A}$. We found that $F_{A}$ is expected to decrease as the population size or mutation rate increase (Fig. 4D), ranging from >90% when the population size is 10,000 or the mutation rate is $6\cdot 10^{-9}$, to about 10% when the population size is above 1,000,000 (less than the experimental population size, which was 6,425,000) or the mutation rate is above $2\cdot 10^{-6}$ (less than the inferred mutation rate, which is $2.965\cdot 10^{-6}$). Thus, our model provides a testable prediction: if the experiment was repeated under a lower population size (via stronger daily dilutions or in a smaller volume) or a lower mutation rate (via a non-mutagenic stress or stress with a smaller target size such as an antifungal drug), then the fraction of the population descending from aneuploid cells would be much higher.

##### Aneuploidy delays rather than facilitates adaptation

An additional interesting result of our study is that aneuploidy increases, rather than decreases, the adaptation time (Fig. 4E). This happens despite the fact that the mean fitness initially increases faster in the presence of aneuploidy (Fig. 4E). Aneuploidy increases adaptation time because once 2n+1 is common, selection for the mutant strain ($2n+1^{*}$ or $2n^{*}$) is weaker compared to when $2n^{*}$ competes directly with 2n. This is an interesting example of clonal interference (Good et al. 2012) but between fast and slow mutational processes (Kronholm and Collins 2016).

##### Rate and fitness effect of aneuploidy and mutation

We inferred the rates of aneuploidy and mutation and their effects on fitness. We estimate that the aneuploidy rate (i.e. number of chromosome gains per generation) is $1.7\cdot 10^{-3}$, higher than a previous estimate of $6.7\cdot 10^{-6}$ (Zhu et al. 2016). This may be due to genetic instability caused by heat stress (Chen et al. 2012), but we note that there is a general scarcity of empirical data on aneuploidy rates. In addition, we did not find evidence for increased mutation rates in aneuploid cells. Previous empirical studies have suggested that genetic instability (e.g. elevated mutation rates) in aneuploid cells is due to stress associated with the aneuploid state (Bouchonville et al. 2009; Chen et al. 2012; Zhu et al. 2012; Ippolito et al. 2021). However, in the experiment of Yona et al. (2012), both the wild-type and the aneuploid were under heat stress, which may explain why we did not find evidence for an increased mutation rate specifically in aneuploid cells.

##### Effect of ploidy

The evolutionary dynamics may change in haploid yeast, in which aneuploidy results in a second, rather than third, chromosome copy. For example, it has been demonstrated that drug resistance mainly evolves via recessive mutations and aneuploidy in haploid yeast (Soncini et al. 2020), whereas in diploid yeast it evolves via dominant mutations, aneuploidy, and gene/segmental duplications (Barney et al. 2021). Thus, the second chromosome copy of disomic yeast may facilitate further adaptation via duplications, rearrangements, and increased mutational tolerance (Avecilla et al. 2023), while decreasing the chance for adaptation via recessive mutations. Future models and experiments can consider how ploidy and other genomic contexts affect the role of aneuploidy in adaptive evolution.

##### Conclusions

Here, we tested the hypothesis that aneuploid cells are an evolutionary “stepping stone”, or adaptive intermediate, between wild-type euploid cells and mutant euploid cells in the evolutionary experiment of Yona et al. (2012). Our results suggest that, although it seems the population goes from euploid to aneuploid and back, this is not the case at the individual level. We estimate that only about 10–15% of the euploid cells descended from aneuploid cells, whereas the rest are direct descendants of the wild-type euploid cells. Thus, aneuploidy can delay, rather than accelerate, adaptation, and cells that become aneuploid may leave less descendants than cells that remain euploid. This surprising result reinforces the importance of mathematical models when interpreting evolutionary dynamics. Moreover, our study emphasizes the unintuitive outcomes of clonal interference between mechanisms for generation of variation that differ in their rate of formation and distribution of fitness effects, including mutation, copy number variation, horizontal gene transfer, and epigenetic modifications.

## Models and Methods

###  

####  

##### Evolutionary genetic model

We model the evolution of a population of cells using a Wright–Fisher model (Otto and Day 2007), assuming a constant effective population size *N*, non-overlapping generations, and including the effects of natural selection, genetic drift, aneuploidy, and mutation. We focus on beneficial genetic modifications, neglecting the effects of deleterious and neutral mutations or karyotypic changes. The model allows for a single aneuploid karyotype (e.g. chromosome III duplication) and a single mutation to accumulate in the genotype. Thus, the model follows four genotypes (Fig. 1): euploid wild-type, 2n; the initial genotype; euploid mutant, $2n^{*}$, with the standard karyotype and a single beneficial mutation; aneuploid wild-type, 2n+1, with an extra chromosome, i.e. following chromosome duplication; and aneuploid mutant, $2n+1^{*}$, with an extra chromosome and a beneficial mutation.

Transitions between the genotypes occur as follows (Fig. 1): beneficial mutations from 2n to $2n^{*}$ and from 2n+1 to $2n+1^{*}$ occur with probability *μ*, the mutation rate. We neglect back-mutations (i.e. from $2n^{*}$ to 2n and from $2n+1^{*}$ to 2n+1). Aneuploidy is formed by chromosome mis-segregation, so that cells transition from 2n to 2n+1 and from $2n+1^{*}$ to $2n^{*}$ with probability *δ*, the aneuploidy rate. That is, we assume chromosomes are gained and lost at the same rate, and we neglect events that form a less-fit genotype (i.e. 2n+1 to 2n and $2n^{*}$ to $2n+1^{*}$). A model that assumes an increased rate of chromosome loss in aneuploid cells [as in Sheltzer et al. (2011)] did not perform well, probably due to lack of statistical power, and was abandoned.

In the experiment by Yona et al. (2012), the population was grown every day from $1.6\cdot 10^{6}$ cells until reaching stationary phase and then diluted 1:120. Thus, we set the population size to $N=6.425\cdot 10^{6}$, the harmonic mean of $\{2^{k}\cdot 1.6\cdot 10^{6}{\}}_{k=0}^{7}$ (Crow and Kimura 1970). The initial population has *N* cells with genotype 2n. The effect of natural selection on the frequency $f_{i}$ of genotype $i=2n,2n+1,2n+1^{*},\text{or}\;2n^{*}$ is given by


(1) $$f_{i}^{s}=\frac{\;f_{i}w_{i}}{\bar{w}},$$

where $w_{i}$ is the fitness of genotype *i* and $\bar{w}=\sum_{\;j}\;f_{j}w_{j}$ is the population mean fitness. The effect of mutation and aneuploidy on genotype frequencies is given by


(2) $$\begin{array}{rl} f_{2n}^{m} & =(1-\delta -\mu )f_{2n}^{s},\\ f_{2n+1}^{m} & =\delta f_{2n}^{s}+(1-\mu )f_{2n+1}^{s},\\ f_{2n+1^{*}}^{m} & =\mu f_{2n+1}^{s}+(1-\delta )f_{2n+1^{*}}^{s},\\ f_{2n^{*}}^{m} & =\mu f_{2n}^{s}+\delta f_{2n+1^{*}}^{s}+f_{2n^{*}}^{s}. \end{array}$$

Finally, random genetic drift is modeled using a multinomial distribution (Otto and Day 2007):


(3) $$f^{\prime }∼\frac{1}{N}\cdot \mathrm{Mult}(N,\;f^{m}),$$

where $f^{m}=(f_{2n}^{m},\;f_{2n+1}^{m},\;f_{2n+1^{*}}^{m},\;f_{2n^{*}}^{m})$ are the frequencies of the genotypes after mutation and aneuploidy, $f^{\prime }$ are the genotype frequencies in the next generation, and Mult(N,f) is a multinomial distribution parameterized by the population size *N* and the genotype frequencies f. Overall, the change in genotype frequencies from one generation to the next is given by the transformation $f_{i}\to f_{i}^{\prime }$.

##### Empirical data for model inference

We use the results of evolutionary experiments reported by Yona et al. (2012). In their heat-stress experiment, four populations of *S. cerevisiae* evolved under 39 °C. Aneuploidy fixed (frequency >95%) in all four population in the first 450 generations. Hereafter, fixation or elimination of a genotype by generation *t* means that more than 95% or less than 5% of the population carry the genotype at generation *t*, and possibly earlier. In the original analysis of Yona et al. (2012), samples were routinely extracted from the evolving populations and tested for indication of heat-shock tolerance. The first generation in which such indication was found was generation 200. Therefore, we determine that aneuploidy did not reach high frequency before generation 200. The experiment continued with two populations, in which aneuploidy was eliminated by generation 1,700 and 2,350 while still under the same conditions of elevated heat (39 °C).

##### Likelihood function

Because our model, just like the Wright–Fisher model, is non-linear and stochastic, computing the distribution of fixation time T(g) of genotype *g* for use in the likelihood function is intractable (it is even hard to use a diffusion-equation approximation due to the model having multiple genotypes, rather than just two). We overcome this problem by approximating the likelihood using simulations. We simulate 1,000 experiments per parameter vector $\theta =(\mu ,\delta ,w_{2n+1},w_{2n+1^{*}},w_{2n^{*}})$, resulting in a set of simulated observations $\tilde{X}=\{{\tilde{X}}_{i}{\}}_{i=1}^{1000}$. We then compute the approximate likelihood:


(4) $$\begin{array}{rl} L(\theta ) & =P^{4}(200\leq T(2n+1)\leq 450)\\ & \;\cdot [1-P_{\tilde{X}}^{4}(!\{T(2n^{*})<1700\}|200\leq T(2n+1)\leq 450)\\ & \;-P_{\tilde{X}}^{4}(!\{1700<T(2n^{*})<2350\}|200\leq T(2n+1)\leq 450)\\ & \;+P_{\tilde{X}}^{4}(!\{T(2n^{*})<1700\}\wedge !\{1700<T(2n^{*})<2350\}|200\leq T(2n+1)\leq 450)], \end{array}$$

where !{…} is the “logical not” operator, $P^{4}(\ldots )$ is the fourth power of P(…), and all probabilities $P_{\tilde{X}}(\ldots )$ are approximated from the results of the simulations $\tilde{X}$. For example, $P_{\tilde{X}}(!\{T(2n^{*})<1700\}|200\leq T(2n+1)\leq 450)$ is approximated by taking simulations in which 2n+1 fixed (reached >95%) before generation 450 but not before generation 200, and computing the fraction of such simulations in which $2n^{*}$ did not fix by generation 1,700, and hence aneuploidy did not extinct (reach <5%) before generation 1,700. Supplementary fig. S1, Supplementary Material online, compares results with less and more simulated experiments, demonstrating that 1,000 simulations are likely sufficient.

For a model without aneuploidy (that is, when the aneuploidy rate is fixed at zero, δ=0), we disregard the increased expression in chromosome III and the growth advantage measured in generation 450, and focus on the growth advantage measured in later generations, presumably due to a beneficial mutation. Therefore, the likelihood is approximated by


(5) $$\begin{array}{rl} L_{!}(\theta ) & =1-P_{\tilde{X}}^{4}(!\{T(2n^{*})<1700\})\\ & \;-P_{\tilde{X}}^{4}(!\{1700<T(2n^{*})<2350\})\\ & \;+P_{\tilde{X}}^{4}(!\{T(2n^{*})<1700\}\wedge !\{1700<T(2n^{*})<2350\}). \end{array}$$

##### Parameter inference

To infer model parameters, we use ABC-SMC (Sisson et al. 2007) implemented in the pyABC Python package (Klinger et al. 2018, pyabc.readthedocs.io). This approach uses numerical stochastic simulations of the model to infer a posterior distribution over the model parameters. It is a method of likelihood-free, simulation-based inference (Cranmer et al. 2020), that is, for estimating a posterior distribution when a likelihood function cannot be directly computed. It is therefore suitable in our case, in which the likelihood function can only be approximated from simulations, and cannot be directly computed.

The ABC-SMC algorithm employs sequential importance sampling over multiple iterations (Toni et al. 2009; Klinger and Hasenauer 2017; Syga et al. 2021). In iteration *t* of the algorithm, a set of parameter vectors, $\{\theta_{i,t}{\}}_{i=1}^{n_{t}}$, also called *particles*, are constructed in the following way. A proposal particle, $\theta^{*}$, is sampled from a proposal distribution, and is either accepted or rejected, until $n_{t}$ particles are accepted. The number of particles, $n_{t}$, is adapted at every iteration *t* using the adaptive population strategy (Klinger et al. 2018, pyabc.readthedocs.io). For t=0, the proposal particle is sampled from the prior distribution, p(θ). For t>0, the proposal particle is sampled from the particles accepted in the previous iteration, $\{\theta_{i,t-1}{\}}_{i=1}^{n_{t-1}}$, each with a probability relative to its weight $W_{t-1}(\theta_{i,t-1})$ (see below). The proposal particle is then perturbed using a kernel perturbation kernel, $K_{t}(\theta^{*}|\theta )$, where *θ* is the sample from the previous iteration. Then, a set of synthetic observations ${\tilde{X}}^{*}$ is simulated, and the proposal particle $\theta^{*}$ is accepted if its approximate likelihood (equation 4) is high enough, $L(\theta^{*})>1-\epsilon_{t}$ (or more commonly, if $1-L(\theta^{*})<\epsilon_{t}$), where $\epsilon_{t}>0$ is the *acceptance threshold*, as higher values of $\epsilon_{t}$ allow more particles to be accepted. The acceptance threshold $\epsilon_{t}$ is chosen as the median of the 1−L(θ) of the particles accepted in the previous iteration, t−1, and $\epsilon_{0}=0.01$. For each accepted particle $\theta_{i,t}$, a weight $W_{t}(\theta_{i,t})$ is assigned: for t=0, $W_{0}(\theta_{i,0})=1$, and for t>0, $W_{t}(\theta_{i,t})=p(\theta_{i,t})/\sum_{i=1}^{n_{t-1}}W_{t-1}(\theta_{i,t-1})K_{t}(\theta_{i,t},\theta_{i,t-1})$, where p(θ) is the prior density of *θ* and $K_{t}(\theta \prime ,\theta )$ is the probability of a perturbation from *θ* to θ′. $K_{t}(\theta \prime |\theta )$ is a multivariate normal distribution, fitted at iteration *t* to the particles from the previous iteration, $\{\theta_{i,t-1}{\}}_{i=1}^{n_{t-1}}$, and their weights, $\{W(\theta_{i,t-1}){\}}_{i=1}^{n_{t-1}}$.

Acceptance is determined according to the approximate likelihood (equation 4), which has a maximum value of $L_{\max}=0.875$ (giving a minimal value of $\epsilon_{\min}=0.125$). We terminated the inference iterations when the change in ϵ value from one iteration to the next was small. With our standard prior and model, we reached ϵ=0.13 (or L=0.87) after six iterations, with $n_{6}=982$ accepted parameter vectors and effective sample size = 651 (supplementary fig. S2, Supplementary Material online). Running the inference algorithm with different initialization seeds and less or more simulations for approximating the likelihood produced similar posterior distributions (supplementary fig. S1, Supplementary Material online).

After producing a set of weighted particles from the posterior distribution using the above ABC-SMC algorithm, we approximate the posterior using kernel density estimation (KDE) with Gaussian kernels. We truncate the estimated posterior to avoid positive posterior density for values with zero prior density. The MAP estimate is computed as the maximum of the estimated joint posterior density. We then draw 5,000,000 samples from the posterior distribution to compute the HDI and draw 50,000 samples to visualize the posterior distribution with histograms.

##### Model comparison

We examine several versions of our evolutionary models, e.g. without aneuploidy or with increased mutation rate in aneuploid cells, as well as several different prior distributions (see below). To compare these, we plot posterior predictions: for each model we execute 10,000 simulations using the MAP parameter estimates and plot the distributions of time to fixation of $2n^{*}$, one of the key properties of the model likelihood. These plots visualize the fit of each model to the data. Also, for similar models, we plot the marginal and joint posterior distributions of the parameters; if these are similar, we consider the models interchangeable. We validate this by comparing HDI of posterior distributions.

Where posterior plots are very similar and the number of parameters is the same, we use WAIC, or the widely applicable information criterion (Gelman et al. 2013), defined as


(6) $$\begin{array}{c} \mathrm{WAIC}(\theta )=-2\log\;E[L(\theta )]+2V[\log\;L(\theta )], \end{array}$$

where *θ* is a parameter vector, and E[⋅] and V[⋅] are the expectation and variance taken over the posterior distribution, which in practice are approximated using 50,000 samples from the posterior KDE. We validated that upon resampling WAIC values do not significantly change and that differences in WAIC between models are preserved. WAIC values are scaled as a deviance measure: lower values imply higher predictive accuracy.

##### Prior distributions

We used informative prior distributions for $w_{2n+1}$, $w_{2n+1^{*}}$, and $w_{2n^{*}}$ (we set $w_{2n}=1$), which we estimated from growth curves data from mono-culture growth experiments previously reported by Yona et al. (2012, Figs. 3C, 4A, and S2). We used *Curveball*, a method for predicting results of competition experiments from growth curve data (Ram et al. 2019, curveball.yoavram.com). Briefly, *Curveball* takes growth curves of two strains growing separately in mono-culture and predicts how they would grow in a mixed culture, that is, it predicts the results of a competition assay. From these predictions, relative fitness values can be computed. Because *Curveball* uses a maximum-likelihood approach to estimate model parameters, we were able to estimate a distribution of relative fitness values to be used as a prior distribution by sampling 10,000 samples from a truncated multivariate normal distribution defined by the maximum-likelihood covariance matrix (supplementary fig. S3, Supplementary Material online).

We used growth curves of 2n and 2n+1 in 39 °C to estimate an informative prior distribution for $w_{2n+1}$ (supplementary fig. S3D, Supplementary Material online, assuming $w_{2n}=1$). In this prior distribution, we used the same prior for $w_{2n+1^{*}}$ and $w_{2n^{*}}$. To increase computational efficiency, we also assumed $w_{2n^{*}}>w_{2n+1^{*}}>w_{2n+1}>w_{2n}$; running the inference without this assumption produced similar results. See Supporting Material online for an extended informative prior distribution that uses growth curves of $2n^{*}$ and 2n+1 growing in 39 °C; this prior distribution proved to be less useful.

As a control, we tested an uninformative uniform prior with U(1,6), for (i) all $w_{2n+1}$, $w_{2n+1^{*}}$, $w_{2n^{*}}$, or (ii) only for $w_{2n+1^{*}}$, $w_{2n^{*}}$, using the above informative prior for $w_{2n+1}$. In these cases, the inference algorithm failed to converge.

For the mutation rate, *μ*, and aneuploidy rate, *δ*, we used uninformative uniform priors, $\mu ∼U(10^{-9},10^{-5})$ and $\delta ∼U(10^{-6},10^{-2})$. A wider mutation rate prior, $\mu ∼U(10^{-9},10^{-3})$, produced similar results.

## Supplementary Material

msae052_Supplementary_Data
