# Supplementary material for: Aneuploidy Can Be an Evolutionary Diversion on the Path to Adaptation
Source: Mol Biol Evol. 2024 Mar 1;41(3):msae052. doi: 10.1093/molbev/msae052 (PMC10951435; doi:10.1093/molbev/msae052)
Supplement: msae052_Supplementary_Data [file msae052_supplementary_data.pdf]

Supplementary Material

Ilia Kohanovski, Martin Pontz, Pétra Vande Zande, Anna Selmecki, Orna Dahan, Yitzhak Pilpel, Avihu H. Yona, Yoav Ram

January 29, 2024

Supplementary Analysis

Sensitivity analysis. Changing a single parameter while keeping the rest fixed at the MAP estimate produces a worse fit to the data (Figure S6). Furthermore, we fitted models with a mutation rate fixed at $\mu = 10^{-5}$, 10^{-6} and 10^{-7}. We inferred similar parameters estimates for the model with $\mu = 10^{-6}$ compared to the model with a free μ parameter, in which the inferred mutation rate is $\mu \approx 3 \cdot 10^{-6}$. Inference assuming $\mu = 10^{-5}$ or $\mu = 10^{-7}$ produced similar estimates except that the estimated aneuploidy rate, δ, was higher, and assuming $\mu = 10^{-7}$, the estimated fitness of $2n+1$ was lower (Figure S7).

Extended informative prior distribution. In an extended informative prior distribution, we used additional growth curves of $2n^*$ (*refined* strain from Yona et al. (2012)) and $2n+1$ in 39 °C to estimate w_{2n^*}/w_{2n+1} (Figure S3H). The same distribution was used for w_{2n^*}/w_{2n+1^*}. Thus, our main informative prior uses a single prior distribution for fitness values of $2n+1$, $2n+1^*$, and $2n^*$, whereas the extended informative prior uses one distribution for $2n+1$, and another distribution for both $2n+1^*$ and $2n^*$.

We estimated the parameters under this extended informative prior. Inference took much longer to run but the posterior distribution seemed to converge, as it did not change much in the final iterations. The posterior predictive plot shows that inference with this extended prior produces a posterior distribution that fails to explain the empirical observations (pink in Figure 3). However, the inferred posterior distribution is considerably narrower (compare Figure 2 and Figure S8) and therefore parameter estimates are less variable. The estimated mutation rate was much lower compared to the main informative prior, with $\mu = 2.474 \cdot 10^{-9}$ $[2.423 \cdot 10^{-9} - 2.612 \cdot 10^{-9}]$. Other parameter estimates are: $\delta = 2.705 \cdot 10^{-3}$ $[2.094 \cdot 10^{-3} - 3.094 \cdot 10^{-3}]$, $w_{2n+1} = 1.022$ $[1.021 - 1.024]$,

$w_{2n+1^*} = 1.052$ [1.05 – 1.054], $w_{2n^*} = 1.053$ [1.051 – 1.055], the latter two being much higher compare to the main informative prior. Notably, the mode of the posterior ratio $w_{2n^*}/w_{2n+1} = 1.0009$ is much lower than the mode of the prior ratio of 1.033 (Figure S3H) and closer to the ratio of 1 that we assume in the main informative prior. Together with the posterior predictive results, we conclude that the main informative prior is preferable over the extended informative prior.

Model with transitions to less-fit genotypes We also estimated the parameters of a version of the model that includes transitions (mutation, chromosome loss and gain) to less-fit genotypes (e.g., $2n^*$ to $2n+1^*$),

$$\begin{aligned}
f^m_{2n} &= (1-\delta-\mu)f^s_{2n} + \delta f^s_{2n+1} + \mu f^s_{2n^*} \,, \\
f^m_{2n+1} &= \delta f^s_{2n} + (1-\delta-\mu)f^s_{2n+1} + \mu f^s_{2n+1^*} \,, \\
f^m_{2n+1^*} &= \mu f^s_{2n+1} + (1-\delta-\mu)f^s_{2n+1^*} + \delta f^s_{2n^*} \,, \\
f^m_{2n^*} &= \mu f^s_{2n} + \delta f^s_{2n+1^*} + (1-\delta-\mu)f^s_{2n^*} \,.
\end{aligned} \tag{1}$$

The inferred values are slightly different. The estimated mutation rate, $\mu = 1.036 \cdot 10^{-7}$ [$8.01 \cdot 10^{-8} - 1.339 \cdot 10^{-7}$], corresponds to a mutation target size of $\sim 300 - 500$, assuming the mutation rate per base pair is roughly $2 \cdot 10^{-10}$ (Zhu et al., 2014) or $3.3 \cdot 10^{-10}$ (Lynch et al., 2008). The estimated aneuploidy rate, $\delta = 2.358 \cdot 10^{-4}$ [$1.766 \cdot 10^{-4} - 2.837 \cdot 10^{-4}$] is 5-35-fold higher than in previous studies: for Chromosome III in diploid *Saccharomyces cerevisiae*, Zhu et al. (2014) estimated $6.7 \cdot 10^{-6}$ chromosome gain events per generation, and Kumaran et al. (2013) estimate $3.0 - 4.3 \cdot 10^{-5}$ chromosome loss events per generation (95% confidence interval). The estimated fitness values are $w_{2n+1} = 1.024$ [1.023 – 1.025], $w_{2n+1^*} = 1.025$ [1.024 – 1.026], $w_{2n^*} = 1.032$ [1.031 – 1.033], all relative to the fitness of $2n$, which is set to $w_{2n} = 1$.

We simulated genotype frequency dynamics using parameter samples from the posterior distribution, and computed the posterior distribution of F_A. The mean F_A in this case is just 0.0189 [0.0004 - 0.1214 95% CI], lower than without the transitions to less-fit genotypes. Here, F_A is the sum of frequencies of both $2n^*_A$ and $2n+1^*$, which reaches a frequency of 0.0007. Out of 100,000 posterior samples, none had F_A above 0.05 (i.e., 5% of the population).

References

Kass, R. E. and Raftery, A. E. (1995), ‘Bayes factors’, *J. Am. Stat. Assoc.* **90**(430), 773.

Kumaran, R., Yang, S.-Y. and Leu, J.-Y. (2013), ‘Characterization of chromosome stability in diploid, polyploid and hybrid yeast cells’, *PLoS ONE* **8**(7), e68094.

Lynch, M., Sung, W., Morris, K., Coffey, N., Landry, C. R., Dopman, E. B., Dickinson, W. J., Okamoto, K., Kulkarni, S., Hartl, D. L. and Thomas, W. K. (2008), ‘A genome-wide view of the spectrum of spontaneous mutations in yeast’, *Proceedings of the National Academy of Sciences* **105**(27), 9272–9277.

Ram, Y., Dellus-Gur, E., Bibi, M., Karkare, K., Obolski, U., Feldman, M. W., Cooper, T. F., Berman, J. and Hadany, L. (2019), ‘Predicting microbial growth in a mixed culture from growth curve data’, *Proceedings of the National Academy of Sciences* **116**(29), 14698–14707.

Yona, A. H., Manor, Y. S., Herbst, R. H., Romano, G. H., Mitchell, A., Kupiec, M., Pilpel, Y. and Dahan, O. (2012), ‘Chromosomal duplication is a transient evolutionary solution to stress.’, *Proceedings of the National Academy of Sciences* **109**(51), 21010–5.

Zhu, Y. O., Siegal, M. L., Hall, D. W. and Petrov, D. A. (2014), ‘Precise estimates of mutation rate and spectrum in yeast’, *Proceedings of the National Academy of Sciences* **111**(22), E2310–E2318.

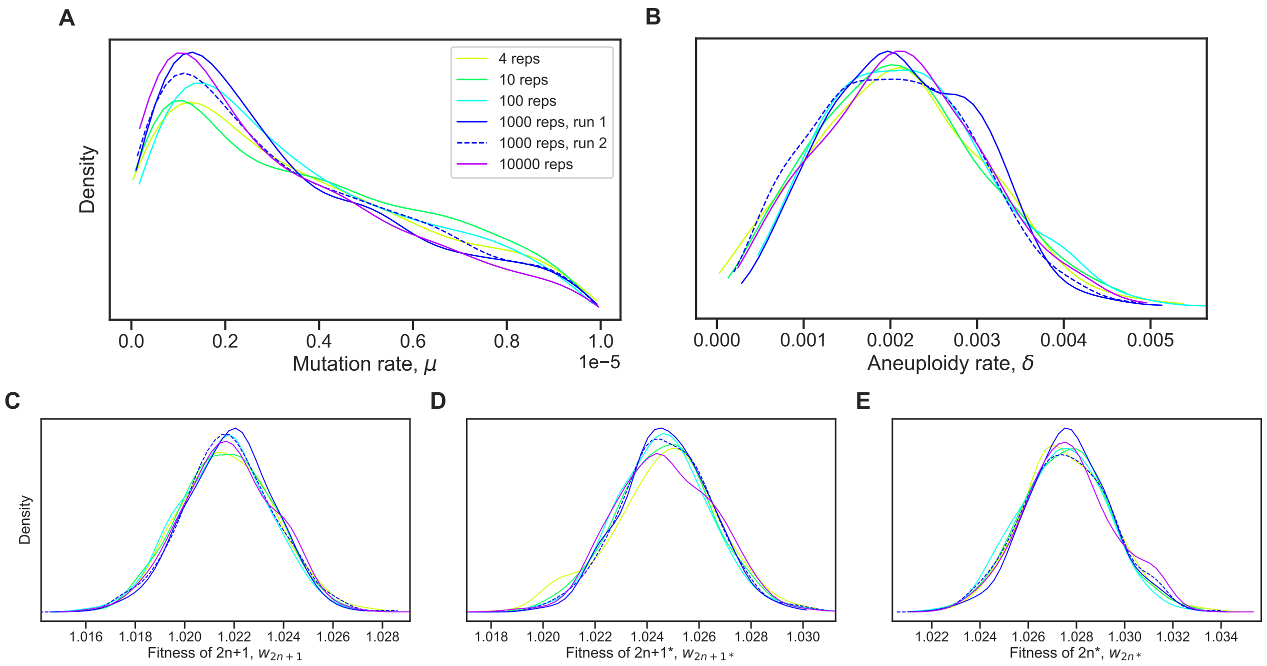

Figure S1: Posterior distribution validation. The posterior distribution of model parameters is roughly the same regardless of the number of simulations (4-10,000 replicates) used to approximate the likelihood (eq. 4).

Table S1: WAIC values for different τ values. Differences of less than 6 are considered of weak significance (Kass and Raftery, 1995).

Model	WAIC
$\tau = 1$	-9
$\tau = 33/32$	-9
$\tau = 2$	-8
$\tau = 5$	-12
$\tau = 10$	-9
$\tau = 100$	-12

WAIC defined in eq. 6.

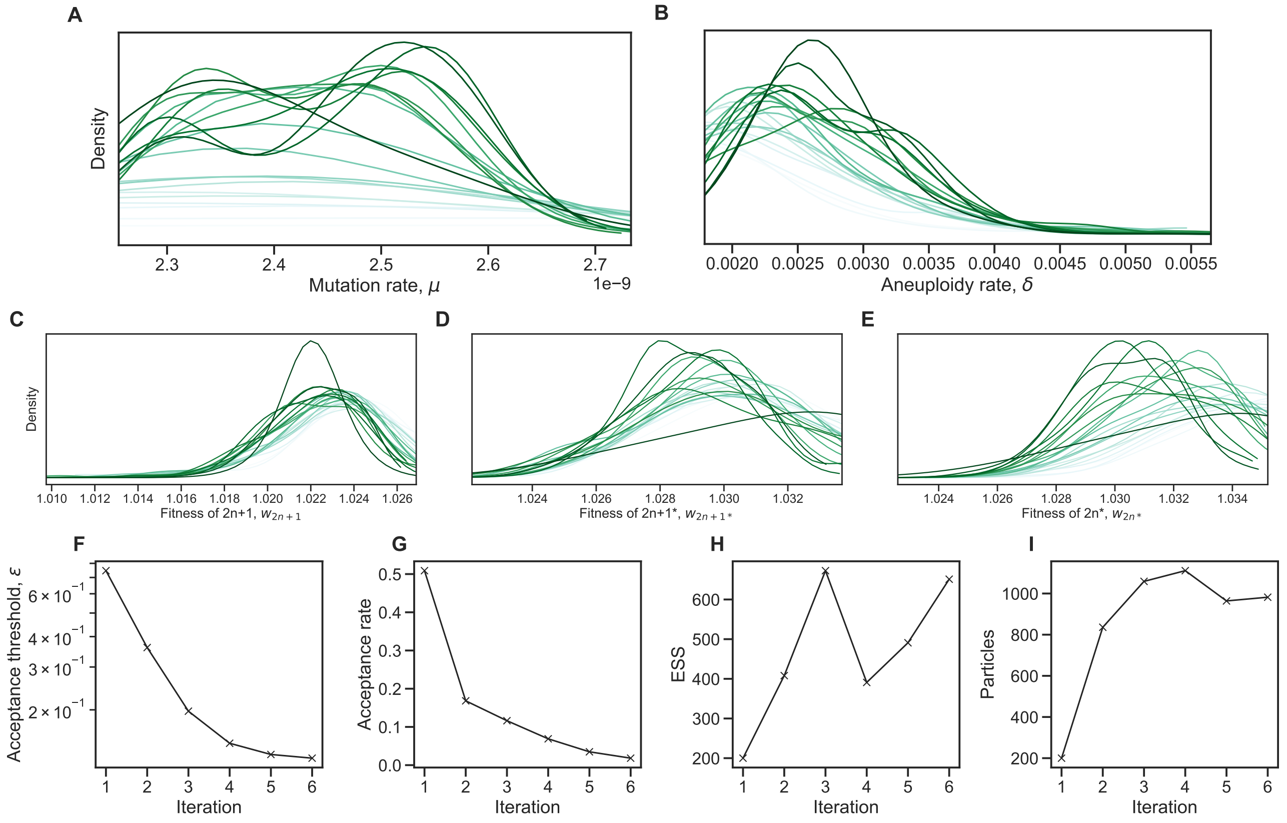

Figure S2: Inference convergence. The ABC-SMC algorithm was used to infer the model parameters. **(A-E)** The approximate posterior distributions of model parameters at each iteration of the ABC-SMC algorithm demonstrates convergence, as the posterior did not significantly change after the first iteration, $t = 1$. **(F-I)** ABC-SMC measures of convergence. After iteration number 6, the acceptance threshold was $\epsilon = 0.13$ (i.e., $\mathcal{L} = 0.87$, eq. 4), the acceptance rate was 0.018, the number of particles was 982, and the effective sample size ESS=651.

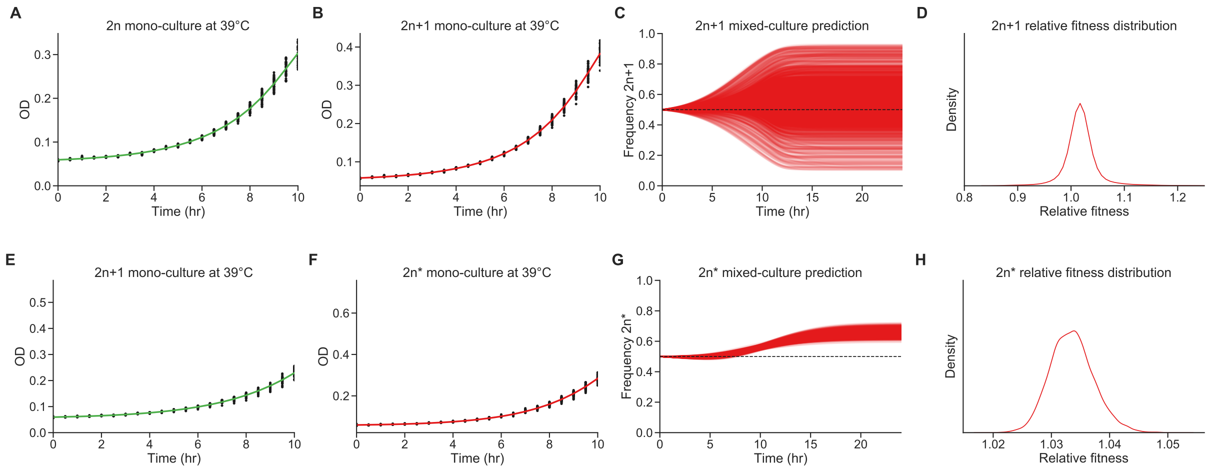

Figure S3: Fitness estimation from growth curves. **(A-D)** Fitness estimation from growth curves of $2n$ and $2n+1$ at 39 °C. w_{2n+1}/w_{2n}=1.024 (95% CI: 0.959 - 1.115). `Curveball` **(E-H)** Fitness estimation from growth curves of $2n+1$ and $2n^*$ at 39 °C. w_{2n^*}/w_{2n+1}=1.033 (95% CI: 1.027 - 1.041). Growth curves previously described in Yona et al. (2012, Figs. 3C, 4A, and S2). Fitness estimated from growth curves using `Curveball`, a method for predicting results of competition experiments from growth curve data (Ram et al., 2019, curveball.yoavram.com). See *Models and Methods, Prior distributions* for more details. **(A,B;E,F)** Mono-culture growth curve data (markers) and best-fit growth models (lines). **(C,G)** The mixed-culture prediction for the strains from A,B and E,F respectively, 6,375 generated curves. **(D,H)** The relative fitness distribution for $2n+1$ relative to $2n$ (panel D) and $2n^*$ relative to $2n+1$ (panel H). Figures generated by `Curveball`.

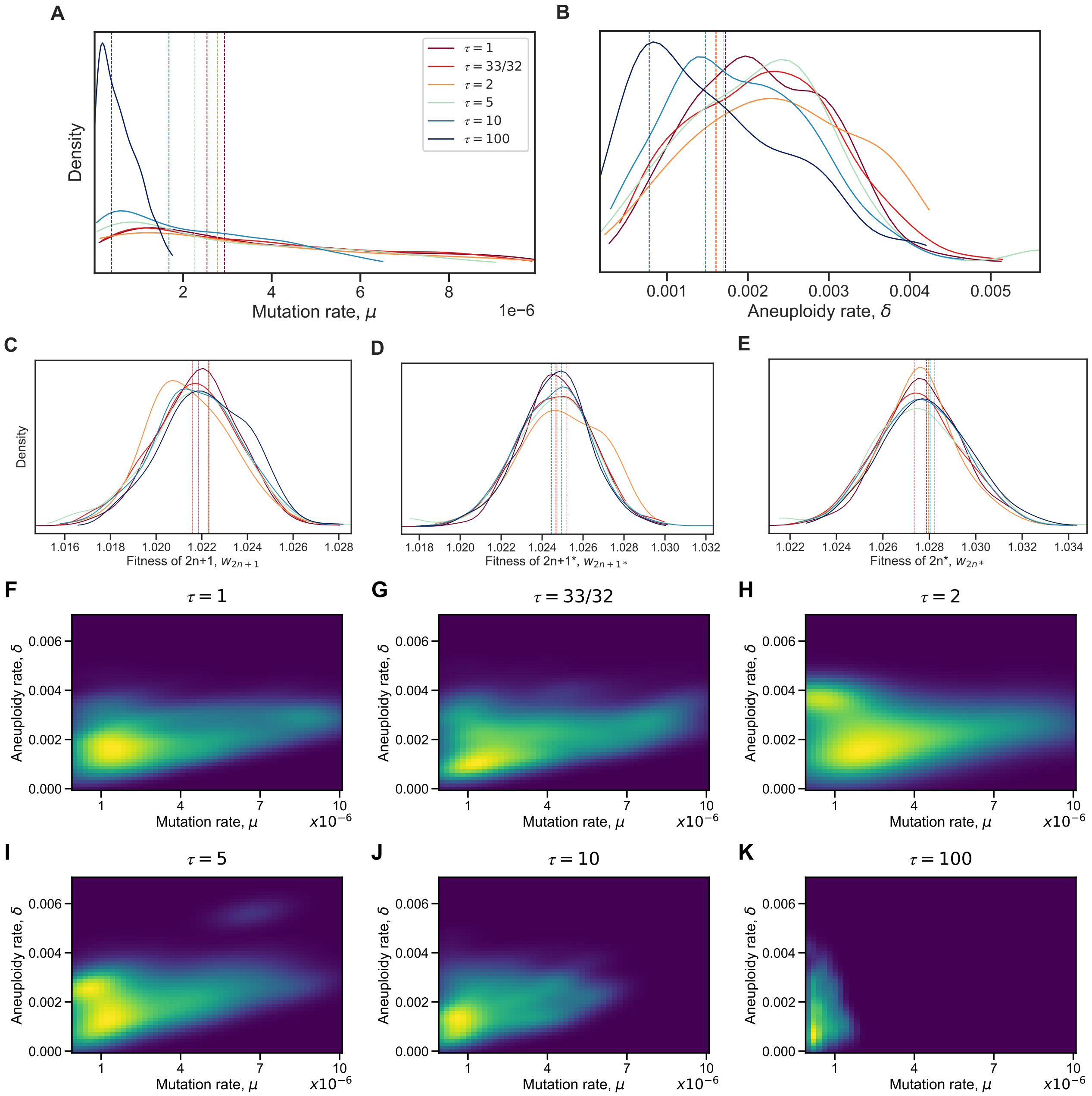

Figure S4: Model with elevated mutation rate in aneuploid cells. (A-E) The inferred posterior distributions for models with different values of τ, the fold-increase in mutation rate in aneuploid cells ($2n + 1$ and $2n + 1^*$). Vertical dashed lines represent the MAP (maximum a posteriori) of each distribution. When the increase in mutation rate is high, $\tau = 10$ and $\tau = 100$, the inferred mutation (A) and aneuploidy (B) rates tend to be lower. **(F-K)** The inferred joint posterior distribution of mutation rate (μ) and aneuploidy rate (δ) with different τ values (dark purple and bright yellow for low and high density, respectively).

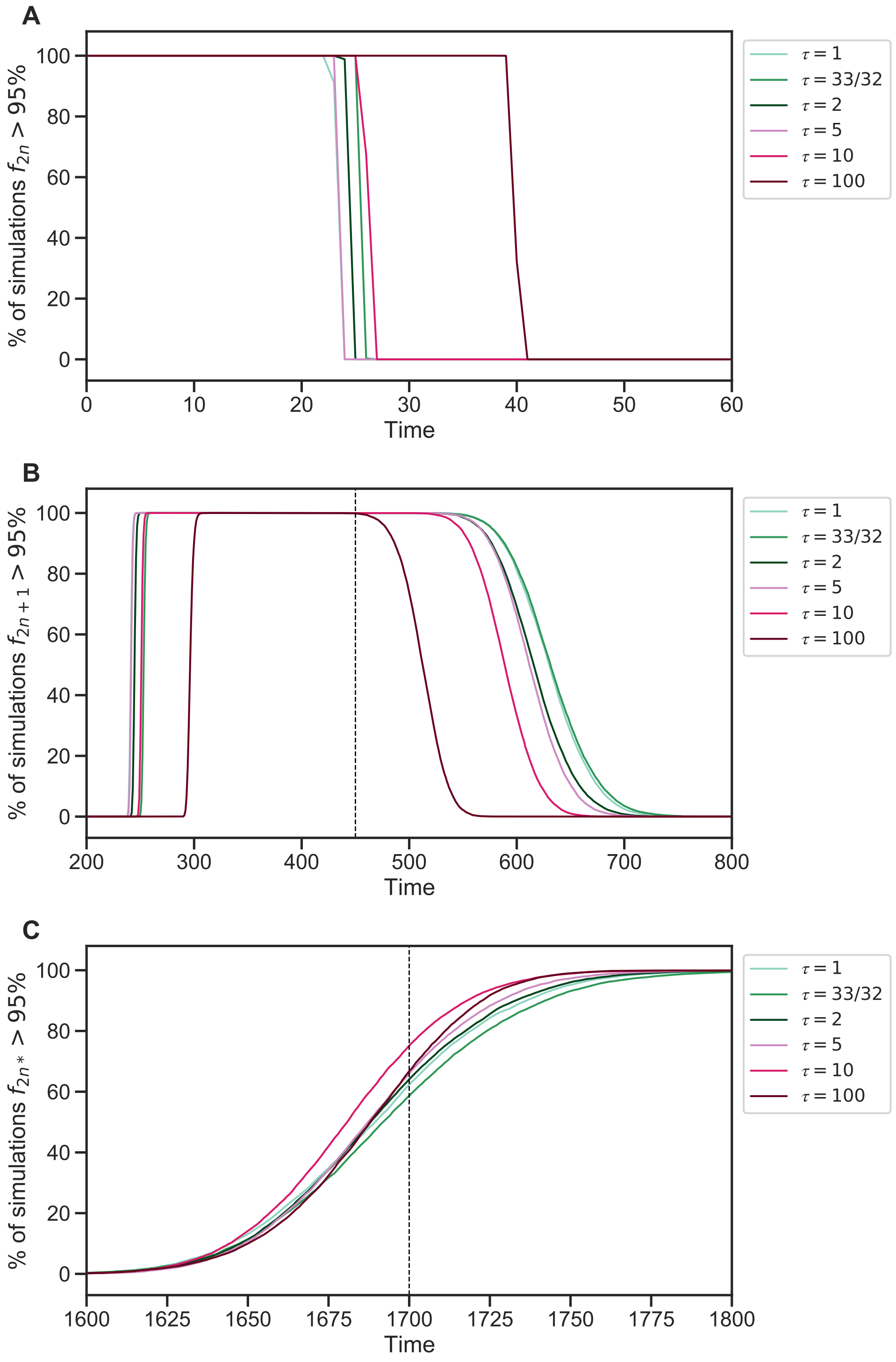

Figure S5: Genotype fixations for models with increased genetic instability. We estimated the parameters for different models, each assuming a different value of τ, the fold-increase in mutation rate in aneuploid cells. We then generated 10,000 simulations using the MAP estimate of each model and evaluated the fraction of simulations in which the frequency of genotype $2n$ (**A**), $2n + 1$ (**B**), and $2n^*$ (**C**) is above 95% (y-axis) at each generation (x-axis). Note that $2n + 1^*$ did not fix. We can see that $\tau = 100$ can be distinguished if the waiting time for $f_{2n} < 95\%$ is known (panel A) or if the waiting time for $f_{2n+1} > 95\%$ or $f_{2n+1} < 95\%$ is known (panel B). It is harder to distinguish between $1 \le \tau \le 10$.

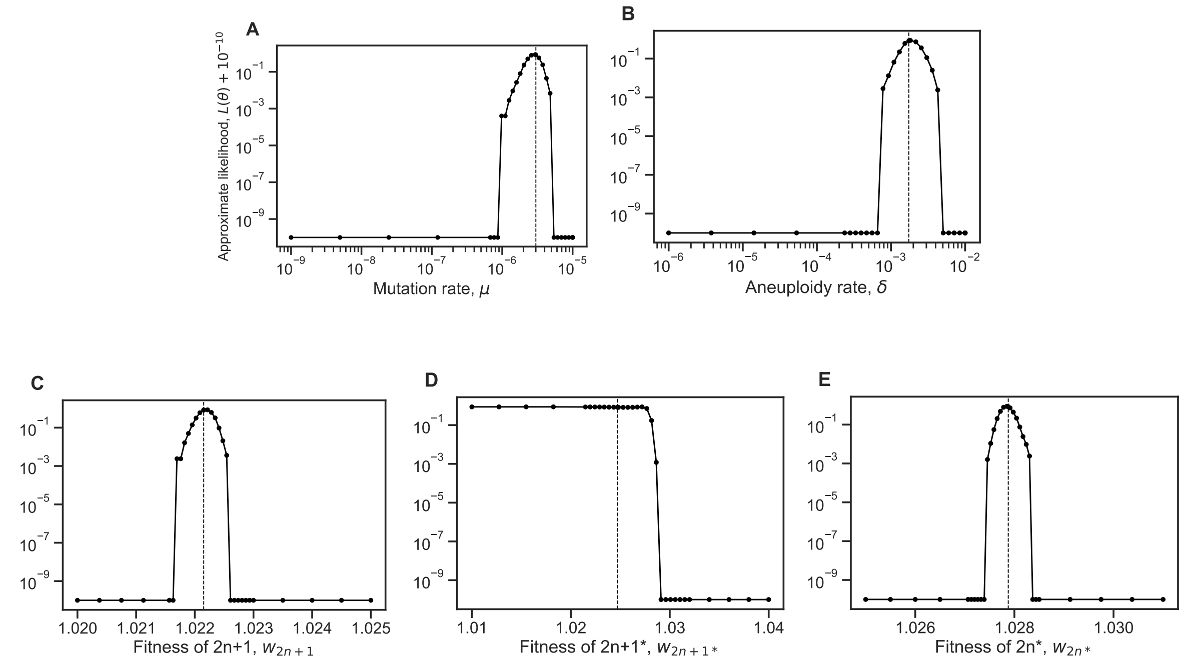

Figure S6: Likelihood profiles. Sensitivity of the model approximate likelihood, $\mathcal{L}(\theta)$, to changing a single parameter while the other parameters remain fixed at their MAP estimates. Dashed vertical line represents the MAP value. The prior distributions for the mutation rate and aneuploidy rate are $\mu \sim U(10^{-9}, 10^{-5})$ and $\delta \sim U(10^{-6}, 10^{-2})$, respectively.

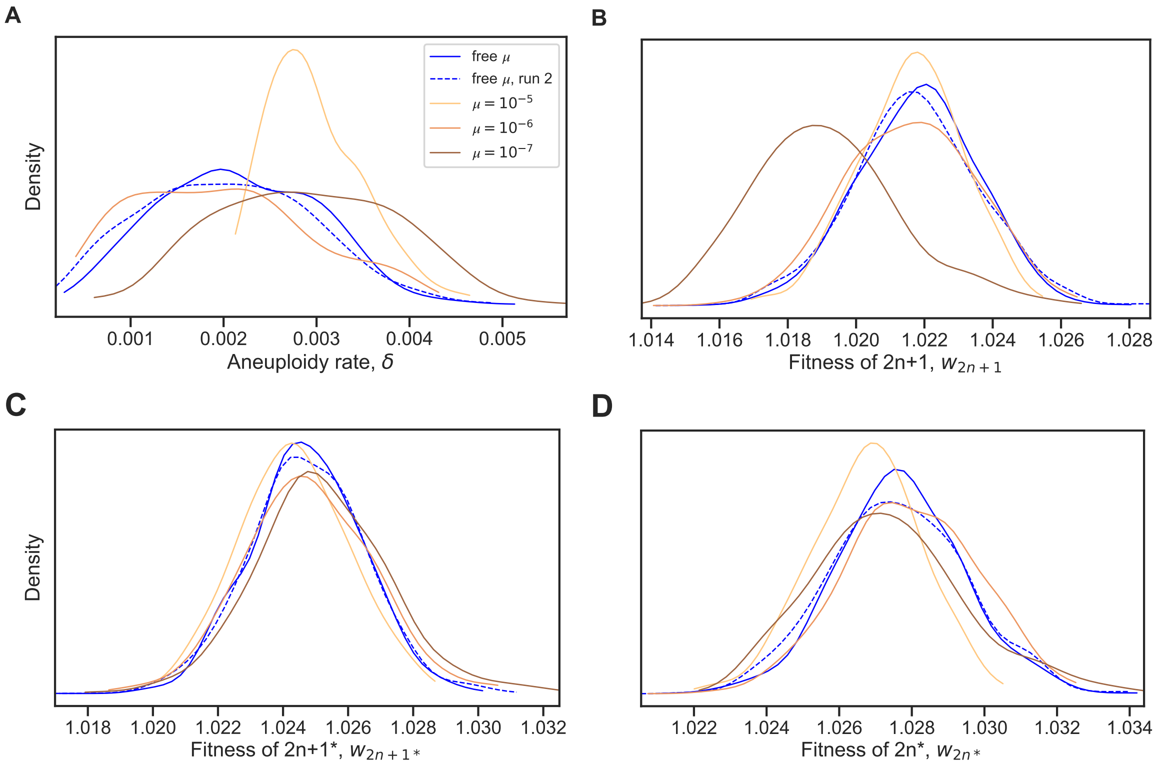

Figure S7: Model with fixed mutation rate. (A-D) The inferred posterior distributions for models with free and fixed mutation rate, μ. The MAP (maximum a posteriori) and 50% HDI (highest density interval) for each model are: **free μ, run 1:** $\delta = 1.720 \cdot 10^{-3}$ $[1.470 \cdot 10^{-3} - 2.786 \cdot 10^{-3}]$, $w_{2n+1} = 1.022$ $[1.021 - 1.023]$, $w_{2n+1^*} = 1.025$ $[1.024 - 1.026]$, $w_{2n^*} = 1.028$ $[1.026 - 1.029]$; **free μ, run 2:** $\delta = 2.129 \cdot 10^{-3}$ $[1.334 \cdot 10^{-3} - 2.695 \cdot 10^{-3}]$, $w_{2n+1} = 1.022$ $[1.02 - 1.023]$, $w_{2n+1^*} = 1.025$ $[1.023 - 1.026]$, $w_{2n^*} = 1.028$ $[1.026 - 1.029]$; **μ = 10^{-5}:** $\delta = 2.903 \cdot 10^{-3}$ $[2.399 \cdot 10^{-3} - 3.156 \cdot 10^{-3}]$, $w_{2n+1} = 1.022$ $[1.021 - 1.023]$, $w_{2n+1^*} = 1.024$ $[1.023 - 1.025]$, $w_{2n^*} = 1.027$ $[1.026 - 1.028]$; **μ = 10^{-6}:** $\delta = 1.917 \cdot 10^{-3}$ $[9.624 \cdot 10^{-4} - 2.447 \cdot 10^{-3}]$, $w_{2n+1} = 1.022$ $[1.02 - 1.023]$, $w_{2n+1^*} = 1.025$ $[1.023 - 1.026]$, $w_{2n^*} = 1.028$ $[1.027 - 1.029]$; **μ = 10^{-7}:** $\delta = 2.901 \cdot 10^{-3}$ $[2.139 \cdot 10^{-3} - 3.671 \cdot 10^{-3}]$, $w_{2n+1} = 1.019$ $[1.017 - 1.02]$, $w_{2n+1^*} = 1.025$ $[1.024 - 1.026]$, $w_{2n^*} = 1.027$ $[1.026 - 1.029]$.

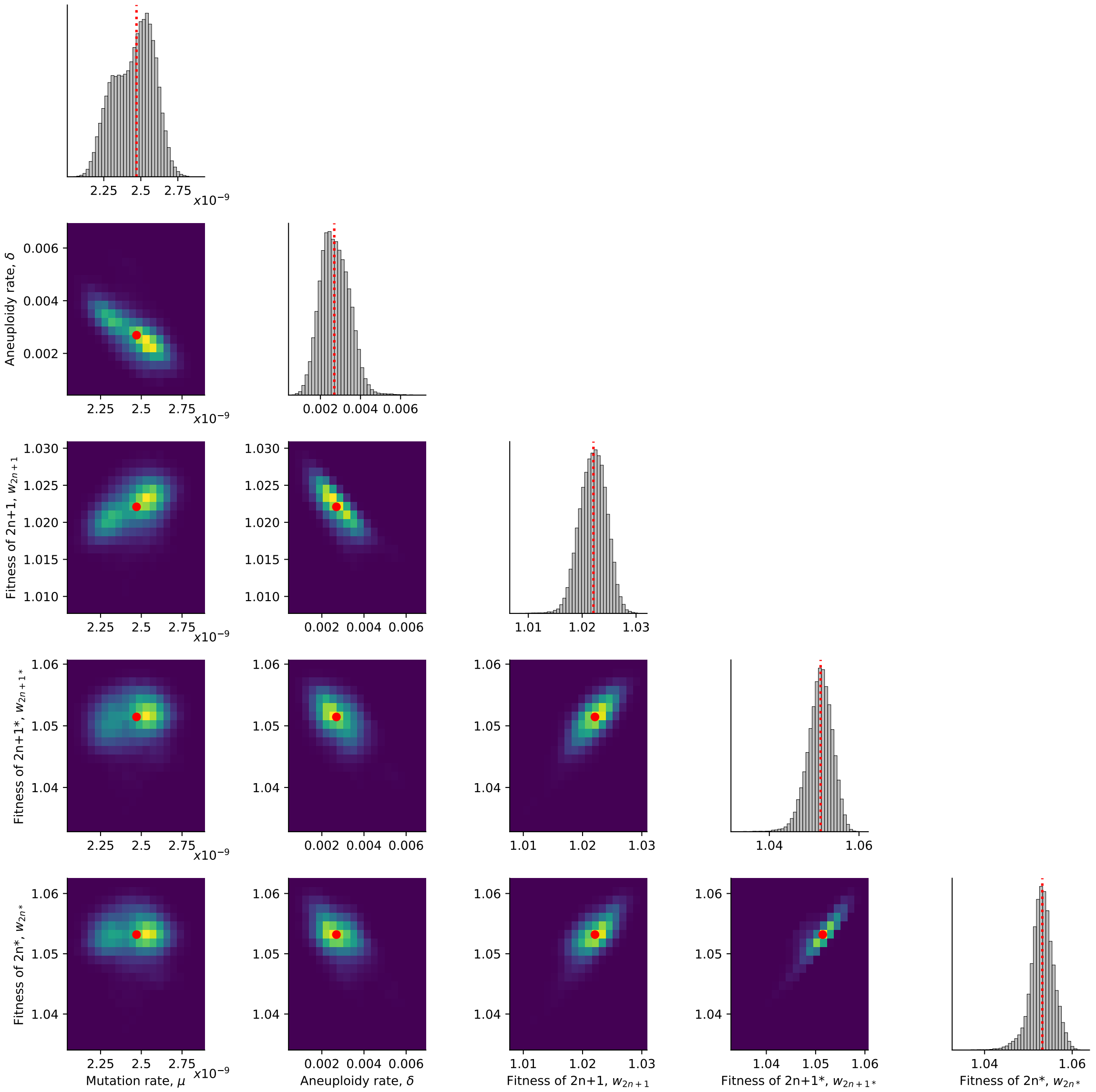

Figure S8: Posterior distribution of parameters inferred with the extended prior distribution. On the diagonal, the inferred posterior distribution of each model parameter. Below the diagonal, the inferred joint posterior distribution of pairs of model parameters (dark purple and bright yellow for low and high density, respectively). Red markers and orange lines for the joint MAP estimate (which may differ from the marginal MAP, as the marginal distribution integrates over all other parameters).

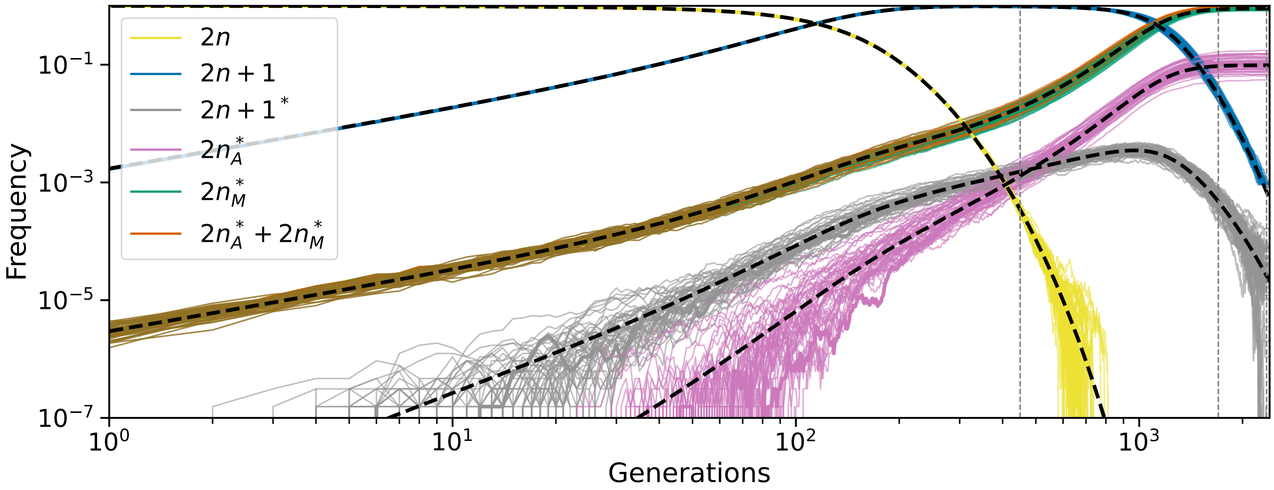

Figure S9: Posterior predicted genotype frequencies in log-log scale. Frequency dynamics of the different genotypes with MAP parameter estimates, same as Figure 4A, but in log-log scale. Black dashed curves for a deterministic model without genetic drift. Clearly, appearance of $2n + 1$ and $2n_M^*$ is deterministic. Appearance of $2n+1^*$, and therefore $2n_A^*$, is stochastic, however, the frequency dynamics are deterministic above a frequency of roughly 0.001. Note that the $2n_M^*$ and the $2n_A^*+2n_M^*$ lines are overlapping for much of their trajectories.

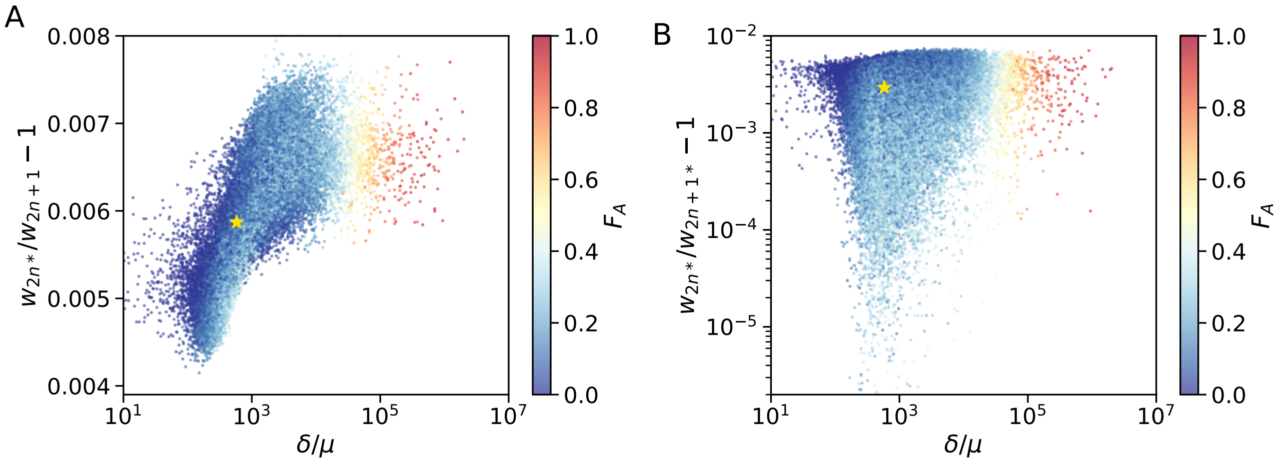

Figure S10: Posterior distribution of F_A. (A,B) F_A values (color coded) as in Figure 4C for different parameter choices on the x- and y-axes. Yellow star shows the MAP estimate.

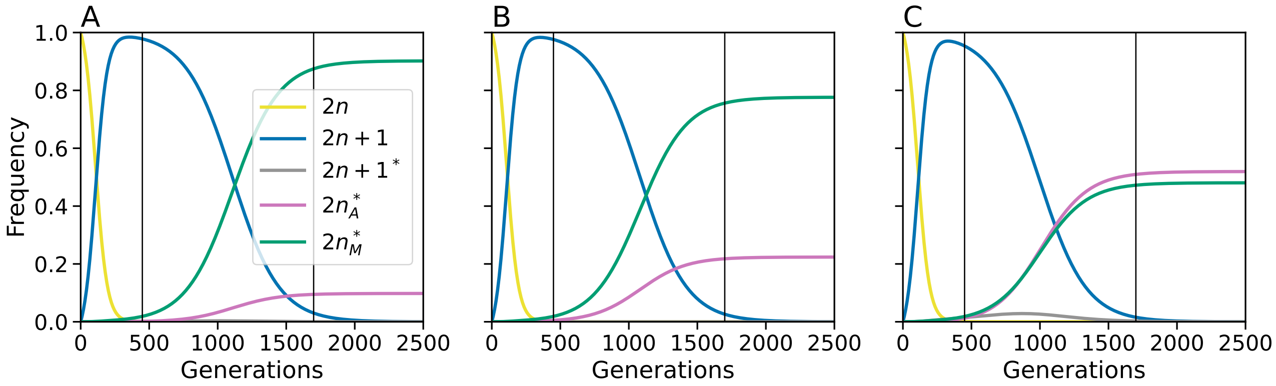

Figure S11: Effect of genomic instability on genotype frequencies. Genotype frequencies in the deterministic model without drift and **(A)** with MAP parameter estimates; **(B)** with 100-fold increase in rate of chromosome loss (transition from $2n + 1^*$ to $2n^*$); or **(C)** with 10-fold increase in mutation rate in aneuploid cells (transition from $2n + 1$ to $2n + 1^*$). Corresponding F_A values (purple line at generation 2,500) are 0.098, 0.223, and 0.519, respectively.
